# Comment on “*Pneumocystis jirovecii* Pneumonia in Patients Treated for Solid Organ Malignancy”

**DOI:** 10.31486/toj.24.0088

**Published:** 2024

**Authors:** Saad Khan, Bilal Ahmad

**Affiliations:** Saidu Medical College, Saidu Sharif, Swat District, Khyber Pakhtunkhwa, Pakistan

## TO THE EDITOR

We recently came across the case series published in the *Ochsner Journal* by Jackson and colleagues,^1^ and the paper really struck a chord with us. This work shines a light on a critical clinical challenge that we think deserves a lot more attention—the management of *Pneumocystis jirovecii* pneumonia (PJP) in patients receiving dose-dense chemotherapy for solid organ malignancies.

As the authors point out, using these intense chemotherapy regimens, such as doxorubicin and cyclophosphamide, has become standard practice for treating early-stage breast cancer. But the downside is that dose-dense chemotherapy seems to be considerably increasing the risk of opportunistic infections such as PJP in these vulnerable patients. And based on the 2 case reports presented by Jackson et al, the consequences can be critical.

The mechanical ventilation and intensive supportive care that these patients required really drives home just how serious PJP can be in this context. Even more concerning is the authors' observation that clear guidelines on PJP prophylaxis are lacking. Current recommendations suggest prophylaxis for groups with a risk >3.5%,^2^ but the 0.6% incidence rate reported by Waks et al suggests that the >3.5% threshold may not be appropriate.^3^

In our view, the existing evidence^3-6^ calls for us to take a hard look at the current guidelines and become much more proactive about PJP prevention in patients on dose-dense chemotherapy. Here are 3 issues we need to focus on:

### Better Data Through Prospective Studies

We need to conduct prospective studies to determine the true incidence of PJP in this patient population, including the impact of concurrent steroid use. Incidence data are crucial for informing new guidelines.

### Tailored Prophylaxis Guidelines

Based on the findings from those studies, we should develop evidence-based guidelines for PJP prophylaxis that are specifically tailored to patients receiving dose-dense chemotherapy. The risk threshold for starting prophylaxis may need to be lowered.

### Raising Clinician Awareness

Oncologists and infectious disease specialists need to be made much more aware of the heightened PJP risk in these patients. Emphasizing early recognition and treatment could make a big difference.

Addressing these issues is vital for improving outcomes in patients with solid organ malignancies who are receiving dose-dense chemotherapy. We commend Jackson et al for reporting on this important topic and hope their case series spurs further research and clinical guidance in this area.
